# Supplementary material for: Learning curve and surgical outcome of robotic assisted colorectal surgery with ERAS program
Source: Sci Rep. 2022 Nov 29;12:20566. doi: 10.1038/s41598-022-24665-w (PMC9709162; doi:10.1038/s41598-022-24665-w)
Supplement: Supplementary file 2 — Supplementary Information 2. [file 41598_2022_24665_MOESM2_ESM.zip › RAL Raw data-Ñ[▒K/073/HS_ñw╝╨░O▒Kñσ.pdf]

正式摘要(電子簽章：[illegible])

W52 / W52 ☐ 英文標題(注意:無英文姓名-請補病人基本檔!) ☐【讀取】憑證

臺中榮民總醫院

出院病歷摘要

轉入號： 轉出號：

[redacted]

| | |
|---|---|
| (8)流水編號 | |
| (9)入院日期 | 110/04/08 (2021) 直腸外科 W52 - 101 |
| (10)轉科日期 | |
| (11)出院日期 | 110/04/15 (2021) 住院天數計 7 日 |

## 診斷

- 入院 :1. Malignant tumor of A-colon
- 出院 :1. Malignant tumor of A-colon
  - s/p Robot Assisted Colectomy(R) on 2021/04/09

## 主訴

- periodical abdomnal pain with tarry stool since last November

## 病史

- This 45 y/o female has hx of
  1. HTN
  2. DM undermedicatio for 6 years
  According to her, periodical abdomnal pain with tarry stool was noted since last November. She deined body weight loss, apptite or bowel change. Due to the above problem, she visited LMD for help this early March, where colonscope showed A-colon tumor. Biopsy was done and showed A-colon Ca, adenocarcinoma. Thus, she visited CRS OPD for second opinion. Lab showed tumor marker in normal range. Abd CT showed focal wall thickening with wall enhancement of A-colon, compatible with colon cancer.
  Thus, she was admitted for scheduled robotic assisted colectomy.

## 體檢發現

-
  - General appearance :
    - easy looking , conscious clear , without cardiopulmonary distress
    - Vital signs自動帶入date 體溫(BT) 脈搏(P) 呼吸(R) 血壓(BP) 身高(BH) 體重(BW) BMI
      20210408 36.8℃ 88次/分 18次/分 118/83mmHg 164.5cm 53.3kg 19.7
    - Vital signs: B.P. 118/83 mmHg , P.R. 88 /min , R.R. 18 /min , B.T. 36.8 ℃
  - HEENT :
    - Eyes:
      - Conjunctiva : pale: .
      - Sclera : normal
    - ENT: no otorrhea, no rhinorrhea, no erythematous mucosa, no injected tonsil, no deformity
  - Neck: supple, freely movable, trachea midline,no deformity, no engorged jugular vein , no palpable thyroid or lymph node enlargement
  - Chest:
    - Inspection: Grossly normal, symmetric and fully expansion, no accessory muscle used, no operation scar, no spider angiomata, no gynecomastia
    - Palpation: no palpable mass or nodule, no subcutaneous emphysema, no tenderness
    - Ausculation: clear breathing sound
  - Heart:
    - Palpation: no thrill, no heave
    - Percussion: normal heart size
    - Ausculation: regular heart beat, normal S1, S2, no gallop, no murmur
  - Extremities: No deformity, full ranged of motion without limitation, no clubbing finger or nail cyanosis, no pitting edema, no ulcer or pigmentation, no obviou lesion, no varicose vein, no bone, joint or muscle tenderness

### CRS CONDITION

- Karnofsky performance status: (100) Normal, no complaints, no evidence of disease
- PE of Abdomen:
- CEA: 0.785 ng/ml
- Imaging Studies:
- Colonoscope report: .
- Sono , CT of abdomen and chest Films:檢驗項目 :CT ABDOMEN-SURVEY 報告日期 :1100331
  主 訴 :
  Result:Finding:
  Imaging Report Form for Colorectal Carcinoma (AJCC 8th)
  1. Imaging Date: 20210331
  Imaging Modality, Imaging by
  CT scan
  Imaging Protocol: Pre- and post-contrast CT scan of abdomen with IV contrast medium administration & oral contrast medium ingestion
  2. Tumor location / Size
  Location:
  Ascending
  Size:
  Non-measurable
  3. Tumor invasion
  Tumor within colonic or rectal wall
  4. Regional nodal metastasis
  No
  5. Distant metastasis (In this study)
  No or Equivocal
  6. Other findings: >Focal wall thickening with wall enhancement of A-colon is mentioned, compatible with colon cancer.
  >Mild fatty infiltration of liver parenchyma with a cyst(1.5cm) in S4
  >A poor-enhancing lesion(length<2.9cm) is located at anterior wall of uterus. Uterine myoma can not be ruled out. Recommend regular follow up at GYN OPD.
  >A low-density lesion(length<2.8cm) is located at left adnexa. Left ovarian cyst can not be ruled out. Recommend regular follow up at GYN OPD.
  >Small-amount ascites at cul-de-sac of pelvic cavity
  Impression:
  A-colon cancer
- Figures:
  1. colon & rectum

## 手術日期及方法

- 手術日期及方法20210409 1. Robotic (XI) assisted right hemicolectomy
  2. Intraoperative colonoscopy : (-)
  3. Ileo-colic artery, right colic artery and right branch of middle colic artery were all ligated via robotic endoclips.
  4. Take down from the hepatic flexure : (+)
  5. OpenGIA (80-3.8) X2 were applied to complete the side to side anastomosis
  6. Cut margin: proximal (ileum) 5 cm, (colon) 3 cm, distal 5 cm.
  7. No drainage tube was inserted
  8. ADHESION BARRIER FILM was placed below the fascia of the para-umbilicus wound

## 住院治療經過

- After admission, pre-op survey was done and there was no contraindication for operation. Robot Assisted Colectomy(R) was perfpormed smoothly on 2021/04/09 and she tolerated it. After OP, she recovered well and no post-op complication was noted. Oral feeding was smooth since 4/10. Under the stable condition, she was discharged and would be followed-up at OPD.

## 合併症

- nil

## 一般檢查

- 累積報告：

| DATE | TIME | ALB | TP | BIL.T | BIL.D | ALKP | AST | ALT | LDH | CHOL | TG | HDL-C | LDL | UA | NH3 | AMY |
|---|---|---|---|---|---|---|---|---|---|---|---|---|---|---|---|---|
| 1100410 | 0237 | 3.7 | | 0.7 | 0.1 | 43 | 19 | 15 | | | | | | | | |

| DATE | TIME | WBC | RBC | HGB | HCT | MCV | PLT | BLST | NEUT | BAND | SEG | LYM | MON | EOS | BAS |
|---|---|---|---|---|---|---|---|---|---|---|---|---|---|---|---|
| 1100408 | 1918 | 5900 | 3.47 | 8.4 | 28.1 | | | | | | | | | | |
| 1100410 | 0237 | 11420 | | 9.6 | 31.1 | | 495 | | 92.1 | | | 6.0 | 1.7 | 0.0 | 0.2 |

| DATE | TIME | NA | K | CL | CA | BUN | CREAT | Mg | P | CRP | ESR | PCT | LIPASE | LACTATE |
|---|---|---|---|---|---|---|---|---|---|---|---|---|---|---|
| 1100410 | 0237 | 139 | 3.6 | | 8.4 | 6 | 0.59 | | | | | | | |

## 特殊檢查

## 病理報告

- Pending

## 其它

## 出院時情況

- 3.改門診治療

## 出院指示

- 

| 藥名 | 劑量 | 劑量單位 | 用法 | 頻次 | 數量 | 數量單位 | 現況 | 天數 | 單位 |
|---|---|---|---|---|---|---|---|---|---|
| $Ferrum drops 30mL | 6 | DROP | PO | QD | 1 | DROP | TH | 3 | 天 |
| Mag. Oxide tab | 2 | TAB | PO | TID | 84 | TAB | TH | 14 | 天 |
| Ultracet tab | 1 | TAB | PO | Q6HPRN | 20 | TAB | TH | 5 | 天 |

## 出院計劃

主治醫師 2062F 蔣鋒帆 / 住院醫師 2334J 陳勇光

正式摘要(電子簽章：**000000214587043)**

W52 / W52

doctorRank:B
